# Block of nicotinic acetylcholine receptors by philanthotoxins is strongly dependent on their subunit composition

**DOI:** 10.1038/srep38116

**Published:** 2016-11-30

**Authors:** Hamid S. Kachel, Rohit N. Patel, Henrik Franzyk, Ian R. Mellor

**Affiliations:** 1 School of Life Sciences, University of Nottingham, University Park, Nottingham, NG7 2RD, UK; 2 Department of Drug Design and Pharmacology, University of Copenhagen, Jagtvej 162, DK-2100 Copenhagen, Denmark

## Abstract

Philanthotoxin-433 (PhTX-433) is an active component of the venom from the Egyptian digger wasp, *Philanthus triangulum*. PhTX-433 inhibits several excitatory ligand-gated ion channels, and to improve selectivity two synthetic analogues, PhTX-343 and PhTX-12, were developed. Previous work showed a 22-fold selectivity of PhTX-12 over PhTX-343 for embryonic muscle-type nicotinic acetylcholine receptors (nAChRs) in TE671 cells. We investigated their inhibition of different neuronal nAChR subunit combinations as well as of embryonic muscle receptors expressed in Xenopus oocytes. Whole-cell currents in response to application of acetylcholine alone or co-applied with PhTX analogue were studied by using two-electrode voltage-clamp. α3β4 nAChRs were most sensitive to PhTX-343 (IC_50_ = 12 nM at −80 mV) with α4β4, α4β2, α3β2, α7 and α1β1γδ being 5, 26, 114, 422 and 992 times less sensitive. In contrast α1β1γδ was most sensitive to PhTX-12 along with α3β4 (IC_50_ values of 100 nM) with α4β4, α4β2, α3β2 and α7 being 3, 3, 26 and 49 times less sensitive. PhTX-343 inhibition was strongly voltage-dependent for all subunit combinations except α7, whereas this was not the case for PhTX-12 for which weak voltage dependence was observed. We conclude that PhTX-343 mainly acts as an open-channel blocker of nAChRs with strong subtype selectivity.

Philanthotoxin-433 (PhTX-433; Fig. 1) is a polyamine-based toxin found in the venom of the Egyptian digger wasp, *Philanthus triangulum*, used to paralyse insect prey by inhibiting nicotinic acetylcholine receptors (nAChRs) and ionotropic glutamate receptors (iGluRs)^1^,^2^. It is structurally characterized by a central tyrosine residue linked via amide bonds to a butyryl chain on one side and to a thermospermine moiety on the other (Fig. 1). This results in a molecule with a relatively bulky and hydrophobic head group and a positively charged (+3) tail at physiological pH. Apart from its inhibitory action on insect nAChRs and iGluRs, PhTX-433 and its closely related synthetic analogue, PhTX-343 (Fig. 1), also exhibit potent activity at vertebrate ionotropic receptors, and their receptor interactions have been quite extensively characterized for mammalian iGluRs, including the AMPA, kainate and NMDA receptor subtypes^3^,^4^,^5^,^6^, as well as for vertebrate muscle-type nAChRs^7^,^8^. These investigations have inferred that philanthotoxins (PhTXs) display some selectivity towards iGluRs over nAChRs.

Based on the observation that in both ionotropic receptor types the inhibition by PhTX-343 is use- and voltage-dependent (i.e. more potent inhibition is observed with increasing negative membrane potentials), it has been proposed that the dominant mode of action involves an open-channel blocking mechanism whereby the polyamine tail penetrates deep into the channel pore where the environment is hydrophilic, while the head group interacts with the more hydrophobic outer parts of the pore^9^,^10^,^11^. In AMPA receptors this is inferred by the observation that receptors lacking the GluA2 subunit are highly sensitive to PhTX-343, whereas those containing GluA2 are almost insensitive^12^. This is due to a single amino acid substitution caused by RNA editing at the so-called “Q/R site” that is located within the pore and forms the selectivity filter^9^.

Strong receptor selectivity was first realized following the development of an analogue in which the two secondary amine functionalities in PhTX-343 (and PhTX-433) were exchanged for methylene groups thereby generating PhTX-12 (Fig. 1). As expected PhTX-12 displayed significantly reduced potency at AMPA receptors and slightly reduced potency at NMDA receptors, but unexpectedly exhibited increased potency at muscle-type nAChRs^5^,^13^. However, the latter finding was associated with a change in mode of action whereby the inhibition was weakly voltage-dependent, remaining strong at positive membrane potentials^8^,^11^.

Interestingly, there is a notable gap in our knowledge of ionotropic receptor inhibition by PhTXs regarding their action on mammalian neuronal-type nAChRs. Only a single study has investigated the effects of PhTX-343 at nAChRs expressed by PC12 cells, showing that it potently antagonised responses to ACh in a voltage-dependent manner^14^.

In the present work, we investigated the inhibitory actions of PhTX-343 and PhTX-12 on some established subtypes of neuronal nAChRs comprising α4β2, α3β4, α7, α4β4 and α3β2, by expression in Xenopus oocytes and voltage clamp recording. Also, we included embryonic muscle-type receptors (α1β1γδ) in our study to facilitate comparison to our previous studies with TE671 cells. We aimed to explore whether PhTXs can be used as subtype-selective inhibitors of nAChRs.

## Materials and Methods

### Reagents and nucleic acids

ACh was from Sigma. PhTX-343 and PhTX-12 were synthesized as described previously^15^.

cDNA clones of rat neuronal nAChR subunits (α3, α4, β2 and β4) and mouse muscle subunits (α1, β1, γ and δ) were from the Salk Institute for Biological Studies (Professor Stephen Heinemann). The human α7 and RIC-3 cDNAs were provided by Professor David Sattelle (University College London). The β2_(V253F)_ and β4_(F255V)_ mutant subunit cDNAs were a kind gift from Dr. Cecilia Borghese, University of Texas at Austin.

Plasmids were linearized and cRNA transcribed using an mMessage mMachine kit (Ambion).

### Xenopus oocyte preparation and injection

Oocytes isolated from mature female *Xenopus laevis* were supplied by the European Xenopus Resource Centre, University of Portsmouth, UK. Oocytes were treated with collagenase (0.5 mg/ml, Sigma type 1 A) in Ca^2+^-free solution (96 mM NaCl, 2 mM KCl, 1 mM MgCl_2_, 5 mM HEPES, 2.5 mM Na-pyruvate, 100 U/mL penicillin, 0.1 mg/mL streptomycin, pH 7.5) with shaking at 19 °C to defolliculate and remove the connective tissue surrounding the cells. After separation, oocytes were washed 7 times with modified Barth’s solution (96 mM NaCl, 2 mM KCl, 1.8 mM CaCl_2_, 1 mM MgCl_2_, 5 mM HEPES, 2.5 mM Na-pyruvate, 0.5 mM theophylline, 50 μg/mL gentamicin, pH 7.5) and kept at 19 °C in the same solution.

Healthy oocytes were injected with cRNA using a Nano-liter Injector (World Precision Instruments Inc, USA). Mixtures of nAChR subunit cRNAs were injected as follows; for heteromeric rat neuronal receptors a 1:1 ratio of α:β at 200 ng/μL; for mouse embryonic muscle a 1:1:1:1 ratio of α:β:γ:δ at 25 ng/μL; human α7 at 100 ng/μL was mixed with RIC-3 at 30 ng/μL. Each oocyte was injected with 50 nL of RNA solution. Injected oocytes were saved in Barth’s solution at 19 °C for two to three days for expression of the target protein. During this time oocytes were regularly checked to remove unhealthy ones.

### Electrophysiology

Electrophysiological recordings were taken from nAChR-expressing oocytes by two-electrode voltage clamp using a Geneclamp 500 voltage clamp amplifier (Axon instruments, USA). An oocyte was placed in the perfusion chamber using a plastic Pasteur pipette and the bath was perfused (~5 mL/min) with fresh Frog Ringer solution (96 mM NaCl, 2 mM KCl, 1.8 mM CaCl_2_ and 5 mM HEPES, pH 7.5). Microelectrodes were pulled from borosilicate glass capillaries (Harvard GC150TF-10) using a programmable micropipette puller (Sutter P97, USA), and they had resistances between 0.5 and 2.5 MΩ when filled with 3 M KCl. The oocyte was voltage-clamped at holding potentials (V_H_) between −60 and −100 mV. ACh was consistently used as the agonist, and it was applied without or together with PhTX analogues via an 8-channel perfusion system (Automate, USA) for 1 min to allow for equilibration of the current. Currents were recorded to a PC via a digidata 1200 analog-to-digital converter (Axon Instuments, USA) using WinEDR v3.2.6 Software (Dr John Dempster, University of Strathclyde, UK).

### Data analysis

WinEDR was used to measure the current amplitude of responses to ACh at the initial peak and at the end of the one-minute application (“late” current). Data were normalized as % control response for PhTX inhibition or % maximum response for ACh agonism. Graphpad Prism 6 was used for data analysis, graph plotting and curve fitting. All plotted points are the mean ± SEM obtained from 4–20 oocytes. Concentration-inhibition and concentration-response curves were used to estimate IC_50_ and EC_50_ values for PhTXs and ACh, respectively, by using the following equations:


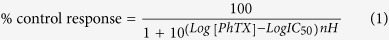



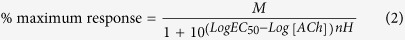


where M is the maximum response and nH is the Hill slope. IC_50_s and EC_50_s were compared for significant differences using an extra sum of squares F-test in Graphpad Prism 6.

## Results

### Sensitivity of nAChR subtypes to ACh

Oocytes expressing each subunit combination were exposed to ACh at concentrations between 10^−8^ M and 10^−3^ M (10^−2^ M for α7) to obtain EC_50_ values (Table S1). Based on peak currents, receptors containing α4 or α1 subunits showed the highest sensitivity to ACh (EC_50_ values of ~10 μM), α3β2 had intermediate sensitivity, while α3β4 and α7 displayed lower sensitivity (EC_50_s > 100 μM). In all cases EC_50_ values were slightly lower (1.4- to 6.7-fold) for late current, but a similar pattern of relative sensitivity emerged. These EC_50_ values demonstrate that, at least for α4β2 and α3β4 where data are available, the dominant subunit stoichiometries are (α4/2)_2_(β2/4)_3_^16^,^17^. Further experiments analysing the effects of PhTX-343 and PhTX-12 on the different nAChR subtypes were conducted by using approximate EC_50_ concentrations of ACh as follows: 10 μM for α4β2, α4β4, and α1β1γδ, 30 μM for α3β2, and 100 μM for α3β4 and α7.

### Subtype selectivity of PhTX-343 and PhTX-12 inhibition

**Figure 2:**
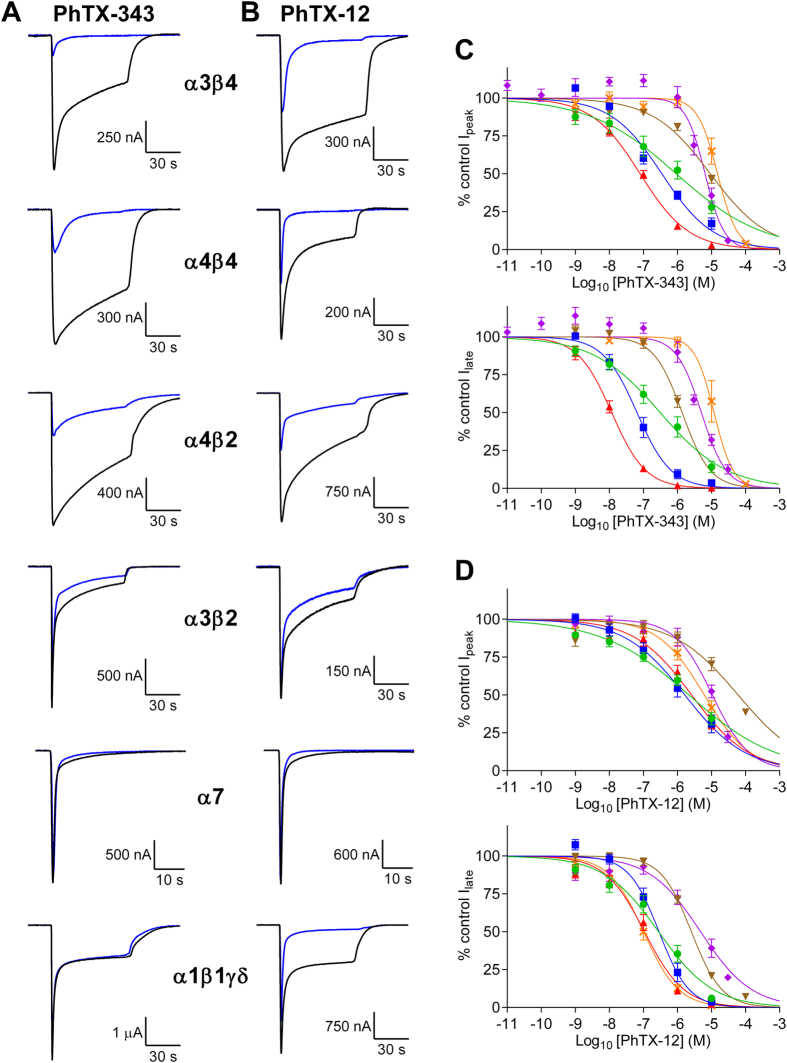
(**A,B**) Responses to ACh in the absence (black) or presence (blue) of 1 μM PhTX-343 (**A**) or PhTX-12 (**B**) for all of the tested nAChR subtypes. (**C,D**) Concentration-inhibition curves for PhTX-343 (**C**) and PhTX-12 (**D**) inhibition of α3β4 (red ▲), α4β4 (blue ■), α4β2 (green ●), α3β2 (brown ▼), α7 (purple ♦) and α1β1γδ (orange **×**) peak (upper) and late (lower) current. The ACh concentrations were 10 μM for α4β4, α4β2 and α1β1γδ, 30 μM for α3β2, and 100 μM for α3β4 and α7. V_H_ = −80 mV. Curves are fitted by Eq. 1 and IC_50_ values are given in Tables 1 and 2. There is a noticeably greater left-right spread of curves for PhTX-343 and a leftward shift for late current inhibition curves as compared to peak current inhibition for both toxins.

*Xenopus* oocytes expressing functional α4β2, α4β4, α3β4, α3β2, α7 and α1β1γδ nAChRs were used to investigate the relative inhibitory potencies of PhTX-343 and PhTX-12. The highest concentration (100 μМ) of each PhTX used in this study was first applied alone (at V_H_ = −80 mV) to oocytes expressing nAChRs, and this did not produce any effect on membrane current. Co-application of PhTX-343 or PhTX-12 with ACh inhibited the current of each subtype of nAChR in a concentration-dependent manner (Fig. 2). Inhibition of the “late” current (1 min after the start of ACh application) was always greater than that of the initial peak current. IC_50_ values for inhibition of each nAChR subtype by PhTX-343 and PhTX-12 are given in Tables 1 and 2, respectively.

**Figure 3:**
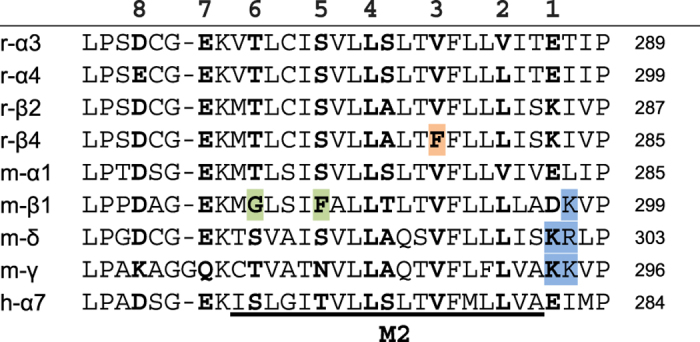
Alignment of amino acid sequences for the M1-M2 loop and M2 region in all of the nAChR subunits used in the present study. Residues expected to line the nAChR pore (termed rings below) are depicted in bold and numbered 1–8 starting at the extracellular terminus. F in ring 3 of r-β4 (a difference that is conserved in other species including humans) is highlighted in orange, F and G in rings 5 and 6 (selectivity filter) of m-β1 are highlighted in green and the positively charged residues at the external mouth of the pore in m-β1, m-δ and m-γ are highlighted in blue.

**Figure 4:**
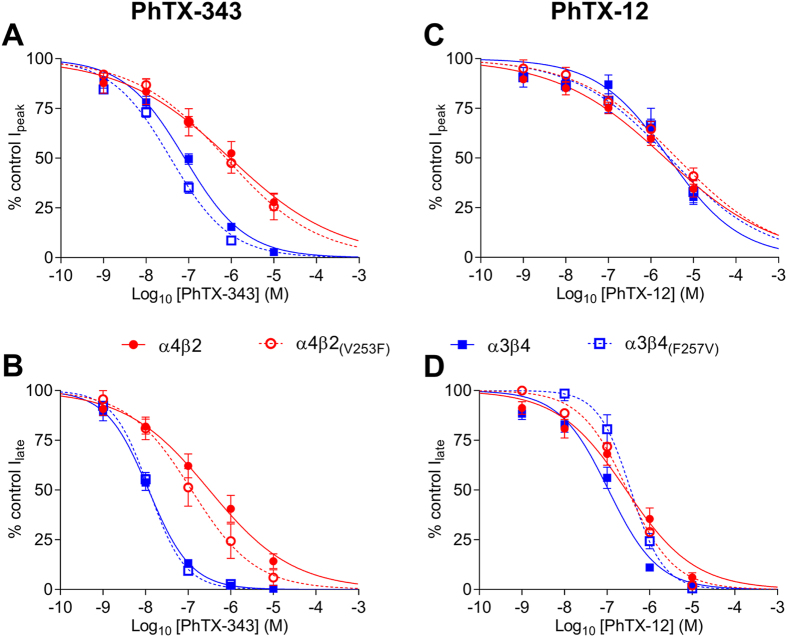
The effect of M2 mutations β2_(V253F)_ and β4_(F255V)_ on PhTX-343 and PhTX-12 inhibition. Concentration-inhibition curves for PhTX-343 (**A**,**B**) and PhTX-12 (**C**,**D**) inhibition of α3β4 (blue ■), α3β4_(F255V)_ (blue □), α4β2 (red ●) and α4β2_(V253F)_ (red ○) peak (**A,C**) and late (**B,D**) current. The ACh concentrations were 10 μM for α4β2 and α4β2_(V253F)_, and 100 μM for α3β4 and α3β4_(F255V)_. V_H_ = −80 mV. Curves are fitted by Eq. 1 and IC_50_ values are given in Tables 1 and 2.

PhTX-343 inhibition of nAChRs seemed to depend mostly on the type of β-subunit present, with receptors containing β4 being most susceptible to the toxin. The ranking by degree of inhibition of ACh responses was: α3β4 > α4β4 > α4β2 > α3β2 > α7 > α1β1γδ with the potency at α3β4 (IC_50_ = 12 nM) being 1000 times higher than at α1β1δγ (IC_50_ = 12 μM) for late current at −80 mV. For PhTX-343 the IC_50_ values for peak and late current inhibition of α4β4 were roughly 3-fold and 5-fold, respectively, lower than those found for α4β2 nAChRs. Likewise, the potency of PhTX-343 on α3β4 nAChRs was higher by 115-fold on peak current and 137-fold on late current as compared to α3β2 (at −80 mV). To investigate the possible cause of this high affinity of PhTX-343 for β4-containing over β2-containing nAChRs, we compared the amino acid sequence of the M2 domains of both β-subunits (Fig. 3), since previous observations indicated that PhTX-343 most likely interacts with this region^11^. This analysis showed only a single amino acid variation within this domain: V at position 253 in β2 being substituted by F at the equivalent position (255; shaded orange in Fig. 3) in β4. To test whether this might be responsible for the observed difference in affinities for PhTX-343 we tested nAChRs containing either β2_(V253F)_ or β4_(F255V)_. As expected, for α4β2_(V253F)_ a 2.6-fold reduction (p = 0.049) in late current IC_50_ was observed as compared to α4β2 (Fig. 4B), but there was no significant change in the peak current value (Fig. 4A). However, we did not see the expected increase in late current IC_50_ value for α3β4_(F255V)_ as compared to that of α3β4 (12 nM at −80 mV in both cases; Fig. 4B), and for the peak current the IC_50_ was even 2.1-fold reduced (p < 0.0001; Fig. 4A). These data suggest some involvement of F255 in β4 in conferring high PhTX-343 affinity, but clearly other factors contribute to PhTX-343 binding. Finally, homomeric α7 nAChRs with no β subunits and muscle-type nAChRs (α1β1γδ) which contain more than one type of non-α subunit, were the least potently inhibited by PhTX-343.

PhTX-12 inhibition of nAChRs did not show any clear pattern of dependence on specific α- or β-subunits. Similar to PhTX-343, PhTX-12 was most potent at α3β4 receptors, and it showed low potency at α7 receptors (lowest potency in this case), but in contrast to PhTX-343 it was as potent at muscle-type receptors (α1β1δγ) as α3β4 receptors. However, the range of PhTX-12 IC_50_ values was narrower than found for PhTX-343; there was only a 49-fold difference in late current IC_50_ value (at −80 mV) between the most potent (0.1 μM for α1β1γδ and α3β4) and the least potent (4.9 μM for α7). The ranking order for nAChR affinity based on late current inhibition by PhTX-12 was: α1β1γδ = α3β4 > α4β2 ≈ α4β4 >> α3β2 > α7.

On the other hand, nAChR subunit combinations showed a slightly different profile for peak current IC_50_ values for PhTX-12. The lowest peak current IC_50_ value at −80 mV, observed for α4β4 nAChRs, was 1.65 μM, increasing to 60 μM for α3β2 nAChRs. The ranking order of potency in this case was: α4β4 > α4β2 > α3β4 > α1β1γδ > α7 >> α3β2. This reflects a strong time/use dependence for inhibition of α1β1γδ, α3β4 and α3β2 by PhTX-12.

We also tested the mutated β2 and β4 subunits in combination with α4 and α3, respectively, for their inhibition by PhTX-12. There were no significant differences in IC_50_ values between α4β2 and α4β2_(V253F)_ for peak or late current inhibition (Fig. 4C,D). For α3β4 and α3β4_(F255V)_ there was no difference between the IC_50_ values for peak current inhibition (Fig. 4C), but for the late current inhibition it was increased by 3.3-fold (p < 0.0001) for α3β4_(F255V)_ (Fig. 4D).

Comparison of the two PhTXs (at −80 mV) with respect to peak current inhibition showed that PhTX-343 was more potent at most of the nAChR subtypes, especially at α3β4 (33-fold), with α1β1δγ being the only exception for which PhTX-12 was slightly more potent. For late current the two toxins were approximately equipotent at α4β2, α3β2 and α7; PhTX-343 was more potent at β4-containing subtypes; PhTX-12 was much more potent at α1β1γδ (119-fold).

### Voltage dependence of nAChR inhibition by PhTX-343 and PhTX-12

For each subunit combination, concentration-inhibition curves were obtained and IC_50_ values determined for both PhTXs at two additional holding potentials (−60 mV and −100 mV as shown in Tables 1 and 2).

**Figure 5:**
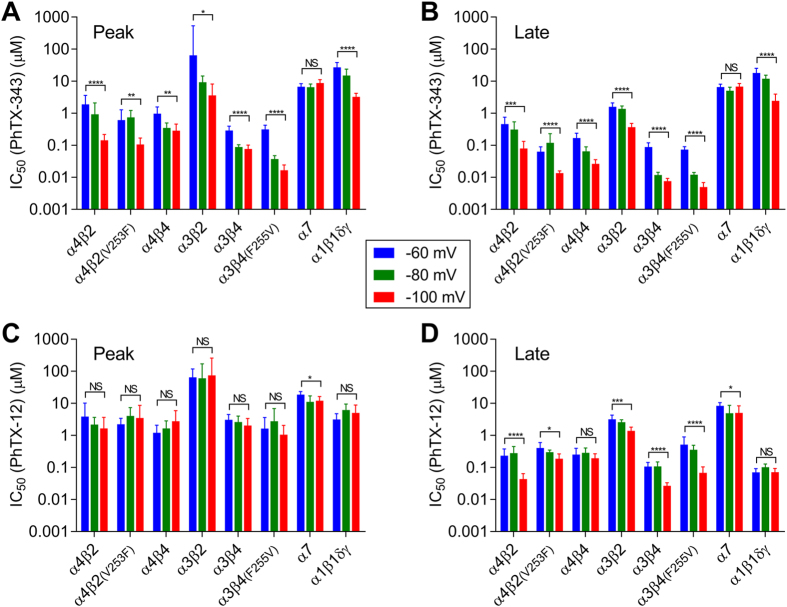
Voltage dependence of inhibition of nAChRs by PhTX-343 (**A,B**) and PhTX-12 (**C,D**) for peak (**A**,**C**) and late (**B**,**D**) current. The bars show IC_50_ (μM) at V_H_ of −60, −80 and −100 mV. ^*^(p < 0.05), ^**^(p < 0.01), ^***^(p < 0.001) and ^***^(p < 0.0001) indicate significant differences in the IC_50_ values for −60 and −100 mV (NS = not significantly different; p > 0.05).

PhTX-343 showed strongly voltage-dependent inhibition of heteromeric nAChRs, but it was voltage-independent for homomeric α7 (Fig. 5). The extent of this depended on the subunits present. It appears that peak current inhibition by PhTX-343 was more sensitive to changes in V_H_ (from −60 mV to −100 mV) on β2-containing nAChRs than on β4-containing receptors, whereas the opposite was found for late current inhibition although this was less pronounced. For example, the peak current IC_50_ values for PhTX-343 inhibition of α4β2 and α3β2 at −60 mV were decreased by 14- and 18-fold, respectively, at V_H_ −100 mV, whereas only a 3.3- and 3.8-fold decrease in IC_50_ was observed for α4β4 and α3β4 nAChRs, respectively.

In contrast to PhTX-343, PhTX-12 inhibition of peak current was voltage-independent for all nAChRs except for homomeric α7 (Fig. 5). On the other hand, based on the late current IC_50_ values there was significant voltage dependence, increasing as follows: α4β2 > α3β4 > α3β2 > α7; but in all cases it was weaker than found for PhTX-343, whereas inhibition was voltage-independent for α4β4 and α1β1γδ (Fig. 5).

### Recovery of nAChRs from inhibition by PhTX-343 or PhTX-12

**Figure 6:**
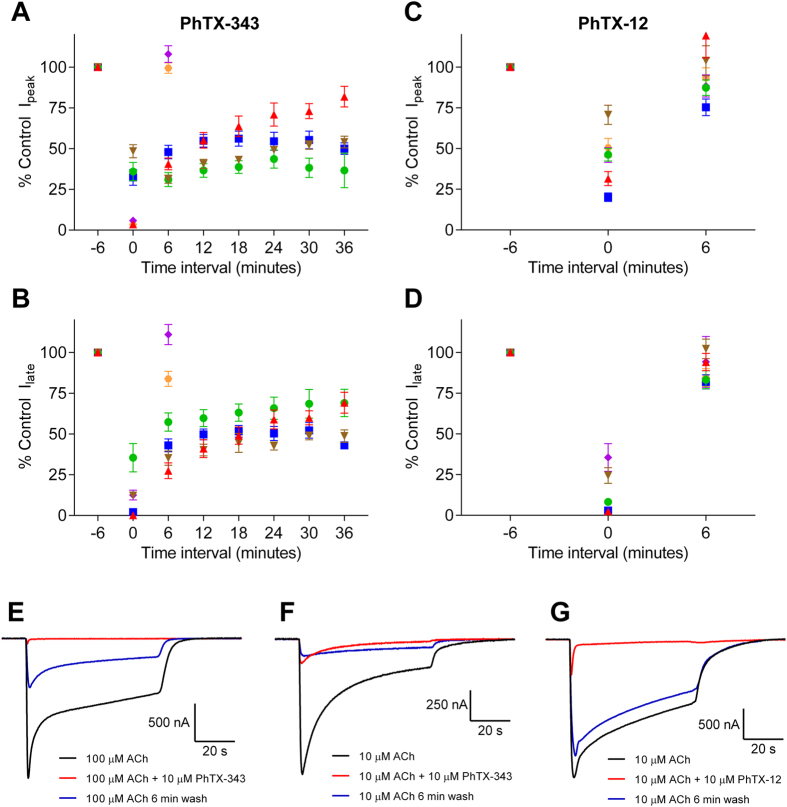
Recovery of responses to ACh following antagonism by PhTX-343 (**A,B**) and PhTX-12 (**C,D**) for peak current (**A**,**C**) and late current (**B**,**D**). A pre-toxin control response to ACh was obtained at −6 min, the response at time 0 was in the presence of PhTX, while the responses from 6 min and onwards were applications of ACh only. ACh was applied at 10 μM for α4β2 (green ●), α4β4 (blue ■) and α1β1γδ (orange 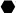), 30 μM for α3β2 (brown ▼) and 100 μM for α3β4 (red ▲) and α7 (purple ♦). PhTX-343 was applied at 10 μM for α4β2, α4β4, α3β4 and α3β2, 30 μM for α7 and 100 μM for α1β1γδ. PhTX-12 was applied at 10 μM for all subunit combinations. (**E**–**G**) Currents in response to ACh before PhTX application (black), ACh co-applied with PhTX (light grey), and ACh alone 6 min after co-application with PhTX (dark grey) for PhTX-343 inhibition of α3β4 (**E**) and α4β2 (**F**), or PhTX-12 inhibition of α4β2 (**G**). V_H_ = −80 mV in all cases.

We investigated the recovery rate of the peak and late current of nAChRs from inhibition by PhTX-343 and PhTX-12 at −80 mV. In all experiments we used approximately the ACh EC_50_ concentration, and following co-application of ACh with PhTX-343 or PhTX-12 we applied ACh alone at 6 min intervals up to 6 times (i.e. 36 min range) (Fig. 6). PhTX-343 was co-applied at 10 μM for α4β2, α4β4, α3β2 and α3β4, at 30 μM for α7, and at 100 μM for α1β1δγ to accommodate the different sensitivities. For all subunit combinations PhTX-12 was applied at 10 μM.

Inhibition of homomeric α7 and muscle-type nAChRs by PhTX-343 was reversible after 6 min washing in frog Ringer solution, while for heteromeric neuronal nAChRs it was irreversible (Fig. 6). For the latter the best recovery of peak current after six ACh applications (36 min) was obtained with α3β4 (to 82% of control), while for α4β2, α4β4 and α3β2 it was very limited or absent. For late current, again α3β4 gave the best recovery (to 69% of control).

Inhibition of nAChRs by PhTX-12 was fully or almost reversible after 6 min. Recovery of the peak current was complete for α3-containing nAChRs, and of the late current for α3β2 and α7. In all other cases 75% (for α4β4 peak current) to 94% (for α3β4 late current) recovery, as compared to the control response, was achieved during the first ACh application after the removal of PhTX-12. Interestingly, the peak current for α3β4 during the first ACh application after removal of PhTX-12 was in fact larger than the control response in 4 out of 6 cells but the combined mean for all cells (119.4% control response, SEM 13.7%) was not significantly greater than control (p = 0.217).

### Inhibition by PhTX-343 and PhTX-12 is largely non-competitive

**Figure 7:**
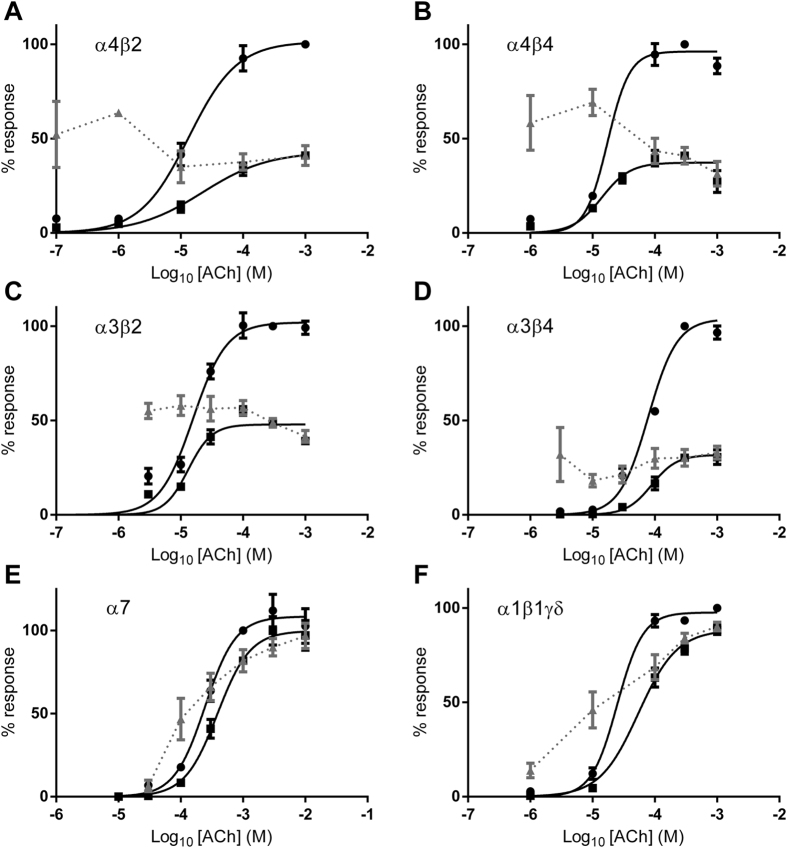
Peak current ACh concentration-response curves in the absence (●) or presence (■) of PhTX-343 at 1 μM for α4β2, α4β4, α3β2 and α3β4, 10 μM for α1β1γδ and 30 μM for α7. Curves were fitted with Eq. 2 and EC_50_ values are given in Table 3. The grey symbols (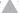) show the % control response for each ACh concentration in the presence of PhTX-343. V_H_ = −80 mV in all cases.

**Figure 8:**
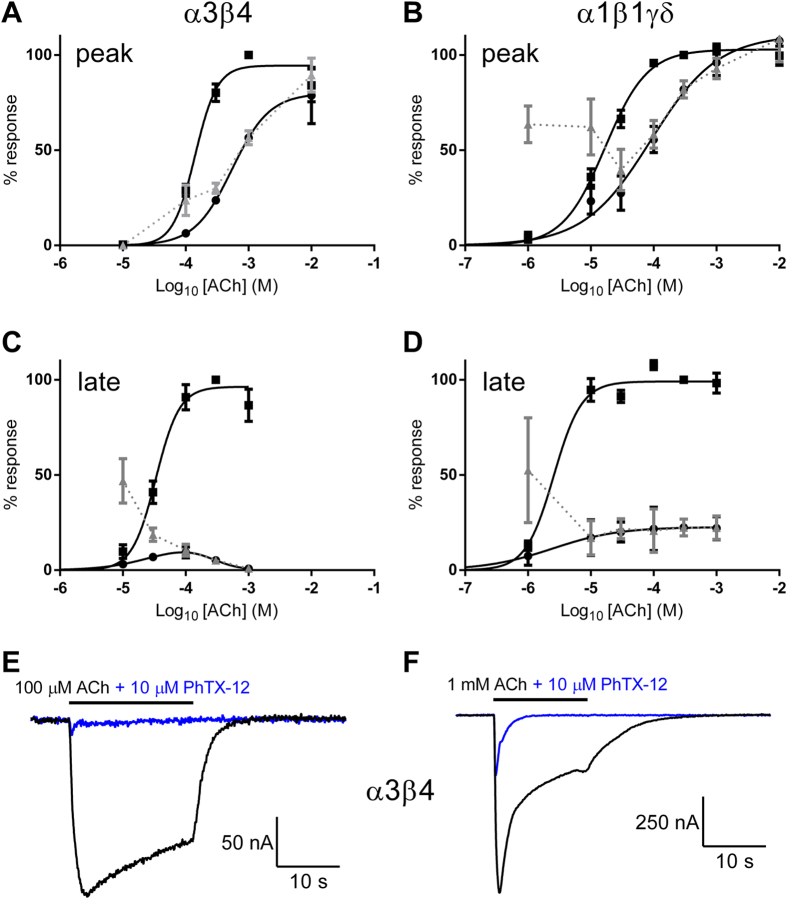
Peak current (A,B) or late current (C,D) ACh concentration-response curves in the absence (●) or presence (■) of PhTX-12 at 10 μM (peak current) or 0.3 μM (late current) for α3β4 and α1β1γδ. Curves were fitted with Eq. 2 and EC_50_ values are given in Table 3. The grey symbols (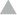) show the % control response for each ACh concentration in the presence of PhTX-12. E-F: α3β4 currents in response to 100 μM (E) or 1 mM ACh in the absence (black) and presence (blue) of 10 μM PhTX-12. V_H_ = −80 mV in all cases.

To further study the mechanism of action by which PhTX-343 and PhTX-12 inhibit acetylcholine-evoked inward current in oocytes expressing nAChRs, we analysed the data obtained from the effects of a single PhTX concentration on acetylcholine concentration-response curves (Figs 7 and 8). The ACh EC_50_ values for peak current in the absence and presence of PhTX-343 for α4β2, α4β4, α3β2, α3β4, α7 and α1β1γδ, as well as for PhTX-12 at α3β4 and α1β1γδ (for which potency was highest) are presented in Table 3.

PhTX-343 (1 μM) caused a reduction of the maximum peak response to ACh, and no change in ACh EC_50_ in all heteromeric neuronal nAChRs was observed (Fig. 7A–D). However, for α7 and α1β1δγ receptors the inhibition of peak current by 30 μM or 10 μM PhTX-343, respectively, was surmountable by increasing ACh concentrations and the ACh EC_50_s were significantly (p < 0.05) increased (Table 3). For late current, the inhibition by PhTX-343 of all nAChRs, including α7 and α1β1γδ, was not surmountable by high ACh concentrations implying that the main inhibitory component was non-competitive.

Inhibition of peak current for both α3β4 and α1β1γδ by 10 μM PhTX-12 was surmountable by higher ACh concentrations, and in both cases the ACh EC_50_ was increased (p < 0.0001) by the toxin (Fig. 8A,B; Table 3). In contrast, the strong inhibition of the late current by 0.3 μM PhTX-12 was insurmountable in both cases (Fig. 8C,D), again implying that the main inhibitory component was non-competitive.

## Discussion

The results presented in this investigation show that the mechanism of inhibition and recovery of nAChRs in response to PhTX-343 is strongly influenced by their subunit combination. The nature of the β-subunit proved to be the main determinant for the inhibitory potency of PhTX-343 on nAChRs, based on our finding that heteromeric β4-containing nAChRs were more sensitive to PhTX-343 than β2-containing receptors. This observation that β4-subunits may have a higher sensitivity than β2-subunits has been noted for other open-channel blockers of nAChRs such as cocaine^18^. On the other hand, nAChR inhibition by PhTX-12 did not depend strongly on the presence of a particular subunit type.

The late current IC_50_ value for α3β4 inhibition by PhTX-343, determined in the present work, was 88 nM at V_H_ = −60 mV. This value is in the same range as reported previously by a patch-clamp study of PC12 cells where an IC_50_ of 100 nM was obtained^14^. It has been shown that PC12 cells might express more than one subtype of neuronal nAChRs but α3β4 combinations are dominant^19^.

Results from our experiments show that inhibition of peak current by PhTX-343 is less effective than inhibition of late current, in agreement with Brier *et al*.^8^ and Liu *et al*.^14^. This variation in potency of PhTX-343 indicates that in order to interact with its binding site and inhibit nAChRs it is a prerequisite that the channel is in its open state^8^. This implies that inhibition occurs by open-channel blockage or by accelerating the desensitization kinetics of the channel. However, the additional voltage dependence of inhibition, demonstrated for all subunit combinations but α7, suggests the former to be most likely. Molecular modelling and photolabile cross-linking studies further support the hypothesis that PhTX-343 binds within the pore of the nAChR^7^,^11^. This is also corroborated by our data obtained from single-residue mutations in the M2 domains of β2 (V253) and β4 (F255). PhTX-343 displayed enhanced potency at the mutant receptor containing β2_(V253F)_, and we interpret this by F providing an improved interaction with the aromatic moiety of PhTX-343 in the shallow part of the pore, while the polyamine moiety retains interactions with the selectivity filter deeper in the pore. However, the inverse mutation (i.e. F255 → V255) in β4 did not reduce the potency so it is likely that other factors, such as channel open-closed kinetics, may play a part. Similar point mutations in the M2 domain of β-subunits have been shown to change the sensitivity of nAChRs to other blockers such as cocaine^18^, substance P^20^, and nitric oxide^21^.

A striking feature of our data was the strong selectivity of PhTX-343 for heteromeric neuronal nAChRs over the muscle-type receptor. It is widely accepted that the pore of the nAChR is lined by eight rings of conserved amino acids which play a role in ion selectivity and conductance. These rings include (from extracellular to intracellular): (1) a charged ring; (2) an outer hydrophobic ring; (3) a valine ring; (4) the equatorial leucine ring (gate); (5) a serine ring; (6) a threonine ring; (7) an intermediate negatively charged ring; and (8) an internal negatively charged ring (Fig. 3). We have previously proposed that PhTX-343 in its extended head-tail conformation can interact with both the valine and equatorial leucine rings via its hydrophobic head group, and with the serine and threonine rings deeper in the pore via its middle and terminal amine groups, respectively^11^. The low sensitivity of muscle-type nAChRs may be due to the presence of phenylalanine and glycine at the serine and threonine positions of rings 5 and 6, respectively, in β1 (shaded green in Fig. 3), thereby reducing the interaction with the polyamine tail of PhTX-343. Additionally, the ratio between positively and negatively charged residues near the extracellular pore entrance may affect the access of PhTX-343 (with a charge of +3) to the pore, and hence to its binding site. Heteromeric neuronal nAChRs will have two or three positively charged amino acids in this area depending on the number of β-subunits (each containing a lysine), while the muscle-type nAChRs have five positively charged residues in this region (1 lysine from β1, 2 lysines from γ, and 1 lysine +1 arginine from δ) (shaded blue in Fig. 3). However, these hypotheses cannot explain the reduced potency of PhTX-343 at the homomeric α7 receptor. Instead this may be as a result of the rapid desensitizing kinetics that significantly reduces the chance of PhTX-343 entering the open pore.

The above proposed mode of binding appears unlikely to have a major impact on PhTX-12 inhibition across the receptors studied here, and indeed PhTX-12 exhibited similar potency at the subunit combinations tested except for α7 and α3β2 receptors that had a lower sensitivity to PhTX-12. These observations may relate to the previous suggestion that PhTX-12 adopts a folded conformation as a result of interaction between the terminal amine and the head region, thereby preventing access to the narrow part of the pore, suggesting binding to an alternative shallow site in the pore resulting in enhanced desensitisation rate^8^,^11^. Amino acid substitutions in this alternative binding region have been shown to influence desensitization rate^22^,^23^. α7 receptors already have fast desensitisation rates so binding of PhTX-12 may not augment this significantly, however, it is not clear why α3β2 displays lower affinity for PhTX-12.

Comparison of the two toxins infers a clear preference for muscle-type nAChR for PhTX-12, while for neuronal nAChR subtypes there are varying degrees of preference for PhTX-343. These results confirm our previous findings that the presence of the secondary amines prevent effective inhibition of muscle-type nAChRs in TE671 cells^5^,^8^,^13^. In the present study, we show that PhTX-12 was 257-fold, 119-fold and 34-fold selective over PhTX-343 on muscle-type nAChRs at V_H_ of −60 mV, −80 mV, and −100 mV, respectively. In comparison, our previous study on human muscle-type nAChRs in TE671 cells showed an 11-fold lower potency of PhTX-12 at this receptor, but a similar 22-fold selectivity over PhTX-343 at −100 mV^8^. It is likely that this difference in potency of PhTX-12 is due to differences in cell type and recording technique used in the two studies.

In the present work, the rate and extent of recovery from inhibition by PhTX-343 and PhTX-12 was contrasting, and it was subunit-dependent for PhTX-343. Heteromeric neuronal nAChRs showed only poor recovery from inhibition by 10 μM PhTX-343 even after six ACh applications over 36 min. Interestingly, α3β4 receptors recovered better than the other three combinations despite being the most potently inhibited, which may be linked to differences in open-closed channel kinetics that offer a higher probability for binding, but also for dissociation or slightly shallower binding due to F255 in the β4-subunit, as also implied by the lower voltage dependence of inhibition. In contrast, muscle-type nAChRs and homomeric α7 receptors showed almost full recovery upon the first ACh application after 6 min, most likely due to weaker interactions between PhTX-343 and the nAChR pore. On the other hand, recovery from inhibition by PhTX-12 was complete or almost complete for all nAChRs upon the first ACh application after 6 min. The enhanced recovery from inhibition by PhTX-12 may reflect the proposed shallower binding site^11^ from which the toxin can more readily dissociate, while poor recovery from blocking by PhTX-343 may be caused by its deep binding site in the pore where it may become ‘entrapped’ during channel closure.

From previous studies, the mechanism of blocking of nAChR inward currents by PhTX analogues has been proposed to be non-competitive. Bixel *et al*.^7^ showed that α-bungarotoxin did not reduce the binding affinity of *N*_*3*_-Ph-PhTX-343-Lys to nAChRs of *Torpedo californica*, and even a slight increase was noticed with carbamoylcholine. Likewise, inhibition of human muscle-type nAChRs by PhTX-343 and PhTX-12 was not influenced by the ACh concentration^8^. Here, our finding that late current inhibition by PhTX-343 and PhTX-12 was not surmountable by increasing the ACh concentration further supports this observation. However, the weaker inhibition of peak current was reduced at high ACh concentrations for PhTX-343 at α7 and α1β1γδ as well as for PhTX-12 at both tested receptors (i.e. α3β4 and α1β1γδ), suggesting that there may be an element of weak competitive inhibition occurring before the stronger non-competitive use-dependent component masks it. An alternative and perhaps more likely explanation is that slower binding of PhTX as compared to that of ACh means that the faster peak current at high ACh concentrations will be much less affected by PhTX.

In conclusion, PhTX-343 potently inhibits ganglionic-type (α3β4) nAChRs with some selectivity over other heteromeric neuronal nAChRs, and with strong selectivity over homomeric α7 and muscle-type nAChRs. In contrast to PhTX-343, PhTX-12 most potently inhibits muscle-type nAChRs but remains a weak inhibitor of homomeric α7 receptors.

## Additional Information

**How to cite this article**: Kachel, H. S. *et al*. Block of nicotinic acetylcholine receptors by philanthotoxins is strongly dependent on their subunit composition. *Sci. Rep.* **6**, 38116; doi: 10.1038/srep38116 (2016).

**Publisher's note:** Springer Nature remains neutral with regard to jurisdictional claims in published maps and institutional affiliations.

## Supplementary Material

Supplementary Table S1

## Figures and Tables

**Figure 1:**
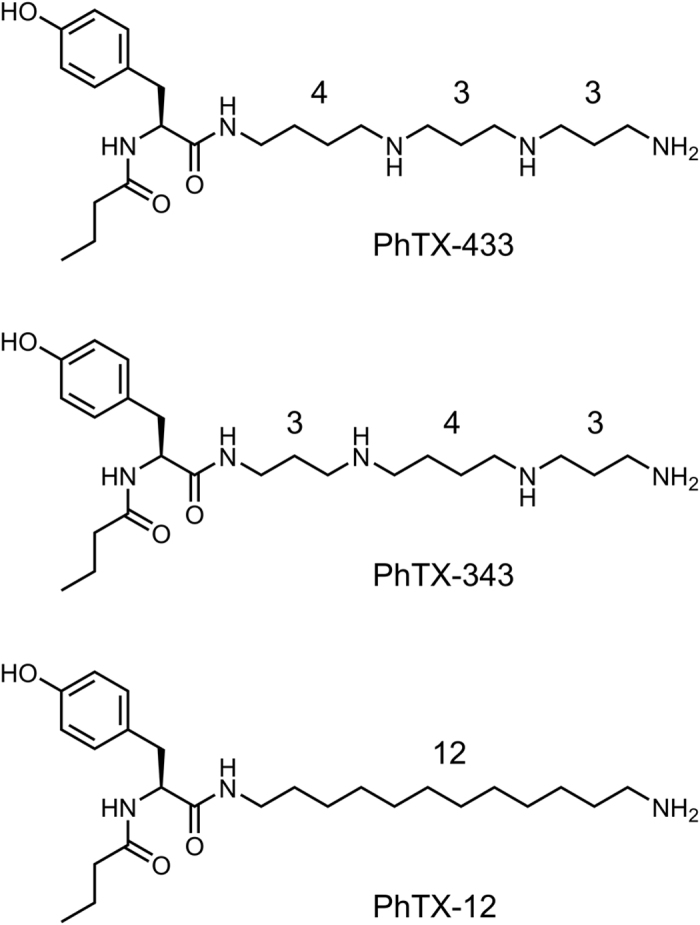
Structures of the naturally occurring PhTX-433 from *Philanthus triangulum* as well as of the two synthetic analogues, PhTX-343 and PhTX-12, used in this study. The numbers indicate the carbon spacing between nitrogen atoms in the polyamine moiety.

**Table 1:** IC_50_ values for PhTX-343 inhibition of nAChR subunit combinations.

	V_H_ (mV)	n	PhTX-343 IC_50_ (95% CI), μM			
			Peak		Late	
α4β2	−60	7	1.90	(1.01–3.60)	0.46	(0.28–0.75)
	−80	7	0.93	(0.41–2.09)	0.31	(0.18–0.54)
	−100	7	0.14	(0.09–0.22)	0.08	(0.049–0.132)
α4β2_(V253F)_	−60	14	0.62	(0.30–1.28)	0.063	(0.045–0.089)
	−80	10	0.75	(0.46–1.23)	0.12	(0.06–0.23)
	−100	8	0.11	(0.07–0.17)	0.014	(0.011–0.016)
α4β4	−60	9	0.97	(0.60–1.58)	0.17	(0.12–0.24)
	−80	9	0.35	(0.24–0.50)	0.065	(0.047–0.089)
	−100	6	0.29	(0.18–0.46)	0.027	(0.020–0.036)
α3β4	−60	7	0.29	(0.21–0.40)	0.088	(0.065–0.120)
	−80	8	0.080	(0.062–0.104)	0.012	(0.010–0.014)
	−100	9	0.077	(0.059–0.102)	0.0077	(0.0063–0.0093)
α3β4_(F255V)_	−60	7	0.31	(0.23–0.42)	0.074	(0.060–0.091)
	−80	9	0.038	(0.029–0.048)	0.012	(0.010–0.014)
	−100	8	0.017	(0.011–0.024)	0.005	(0.0036–0.0068)
α3β2	−60	7	64.5	(7.8–532)	1.59	(1.20–2.11)
	−80	12	9.26	(5.90–14.6)	1.37	(1.12–1.69)
	−100	8	3.59	(1.58–8.19)	0.37	(0.29–0.48)
α7	−60	6	6.71	(5.39–8.35)	6.56†	(5.30–8.12)
	−80	7	6.48	(5.12–8.21)	5.06†	(3.95–6.48)
	−100	9	8.80	(6.92–11.18)	6.77†	(5.44–8.42)
α1β1γδ	−60	8	27.1	(19.1–38.4)	18.0	(12.8–25.4)
	−80	8	15.1	(9.5–24.0)	11.9	(9.2–15.4)
	−100	9	3.26	(2.54–4.17)	2.44	(1.50–3.97)

IC_50_ values for PhTX-343 inhibition of both peak and late current estimated from concentration-inhibition curves in Figs 2 and 4.

^†^For α7 this is based on the area under the current trace as there was no stable late current.

**Table 2:** IC_50_ values for PhTX-12 inhibition of nAChR subunit combinations.

	V_H_ (mV)	n	PhTX-12 IC_50_ (95% CI), μM			
			Peak		Late	
α4β2	−60	8	3.87	(1.47–10.16)	0.23	(0.14–0.38)
	−80	9	2.17	(1.30–3.64)	0.28	(0.17–0.45)
	−100	11	1.64	(0.74–3.67)	0.044	(0.030–0.064)
α4β2_(V253F)_	−60	4	2.22	(1.44–3.42)	0.41	(0.28–0.60)
	−80	5	4.05	(2.22–7.36)	0.3	(0.25–0.35)
	−100	9	3.48	(1.40–8.70)	0.19	(0.13–0.27)
α4β4	−60	9	1.21	(0.70–2.07)	0.25	(0.16–0.40)
	−80	12	1.65	(0.96–2.83)	0.29	(0.21–0.40)
	−100	9	2.77	(1.30–5.92)	0.19	(0.14–0.27)
α3β4	−60	7	3.09	(2.13–4.47)	0.11	(0.08–0.14)
	−80	8	2.62	(1.71–4.01)	0.11	(0.08–0.15)
	−100	8	2.03	(1.22–3.36)	0.027	(0.022–0.033)
α3β4_(F255V)_	−60	5	1.63	(0.74–3.60)	0.52	(0.30–0.90)
	−80	5	2.78	(1.13–6.84)	0.36	(0.26–0.50)
	−100	5	1.05	(0.54–2.05)	0.068	(0.044–0.105)
α3β2	−60	9	65.2	(36.0–118.0)	3.20	(2.39–4.28)
	−80	12	60.6	(21.4–172)	2.57	(2.16–3.06)
	−100	10	74.4	(21.3–259.3)	1.38	(1.05–1.82)
α7	−60	12	18.7	(14.9–23.4)	8.32^†^	(6.55–10.58)
	−80	12	11.1	(7.2–17.1)	4.92†	(2.79–8.66)
	−100	10	12.1	(9.04–16.2)	5.02†	(3.01–8.40)
α1β1γδ	−60	9	3.16	(2.11–4.74)	0.070	(0.056–0.092)
	−80	11	6.18	(4.03–9.46)	0.10	(0.08–0.13)
	−100	8	5.02	(2.86–8.83)	0.071	(0.055–0.093)

IC_50_ values for PhTX-12 inhibition of both peak and late current estimated from concentration-inhibition curves in Figs 2 and 4.

^†^For α7 this is based on the area under the current trace as there was no stable late current.

**Table 3:** ACh EC_50_ values in the absence and presence of PhTX-343 or PhTX-12.

	[PhTX-343]	ACh EC_50_ (95% CI), μM			
		Control		+PhTX-343	
α4β2	1 μM	13	(8.9–20)	22	(5.6–89)
α4β4	1 μM	17	(8.4–36)	14	(7.8–25)
α3β4	1 μM	78	(67–91)	89	(38–207)
α3β2	1 μM	16	(12–20)	13	(9.4–18)
α7	30 μM	245	(198–304)	388^*^	(265–568)
α1β1γδ	10 μM	25	(18–35)	54^*^	(37–80)
	**[PhTX-12]**	**Control**		**+PhTX-12**	
α3β4	10 μM	141	(113–175)	545^****^	(331–897)
α1β1γδ	10 μM	17	(15–20)	91^****^	(48–170)

ACh EC_50_ values estimated from peak current concentration-response curves in the absence and presence of PhTX-343 or PhTX-12 shown in Figs 6 and 7. ^*^p < 0.05 and ^****^p < 0.0001 when compared to the EC_50_ in the absence of toxin by an extra sum of squares F-test.
