# Supplementary material for: Block of nicotinic acetylcholine receptors by philanthotoxins is strongly dependent on their subunit composition
Source: Sci Rep. 2016 Nov 30;6:38116. doi: 10.1038/srep38116 (PMC5128878; doi:10.1038/srep38116)
Supplement: Supplementary Table S1 [file srep38116-s1.pdf]

Supporting Information

Table S1

	n	ACh EC_{50} (95% CI), μM
α4β2	10	11.9 (9.3-15.2)
α4β4	12	9.4 (7.6-11.6)
α3β4	13	103 (96-112)
α3β2	14	17.4 (14.2-21.3)
α7	13	178 (155-205)
α1β1γδ	10	16.0 (13.9-18.3)

Table S1. ACh EC_{50} values for all tested subunit combinations estimated from fitting of Eq. 2 to plots of % maximum peak current vs. ACh concentration.
